#  Study of Mutations in the DNA gyrase *gyrA *Gene of *Escherichia coli *

**Published:** 2010

**Authors:** Razieh Pourahmad Jaktaji, Ehsan Mohiti

**Affiliations:** *Department of Genetics, Faculty of Science, University of Shahrekord, Shahrekord, Iran. *

**Keywords:** Ciprofloxacin, DNA gyrase, *gyrA *Gene, *Escherichia coli*, Mutation

## Abstract

Quinolones are a large and widely consumed class of synthetic drugs. Expanded-spectrum quinolones, like ciprofloxacin are highly effective against Gram-negative bacteria, especially *Escherichia coli*. In *E. coli *the major target for quinolones is DNA gyrase. This enzyme is composed of two subunits, GyrA and GyrB encoding by *gyrA *and *gyrB*, respectively. Mutations in either of these genes cause quinolone resistance. Mutations in QRDR section of *gyrA *are more common in quinolone resistant clinical isolates. However, a mutation outside of this region was also reported. Thus, this study was aimed to provide more information on mutations sites in *gyrA*. For this purpose, spontaneous ciprofloxacin resistant mutants arisen in cultures of *E. coli *ATCC 25922 and MG1655 were isolated on LB agar containing ciprofloxacin. Next, the MICs of these clones were measured and the presence of mutation in *gyrA *was investigated. Results showed that the frequency of ciprofloxacin resistant mutants in cultures of *E. coli *strains was low. However, these mutants had different MICs, depending on the day of isolation. Most of ciprofloxacin-resistant mutants possess mutations in QRDR region and precisely at Ser-83. However, mutations outside of this region were also found at Tyr-50 and Ala-119. In conclusion, the presence of mutations at amino acids 50 and 119 suggests that in addition to QRDR section and Tyr-122, these sites are also essential for DNA gyrase activity.

## Introduction

**Figure 1:**
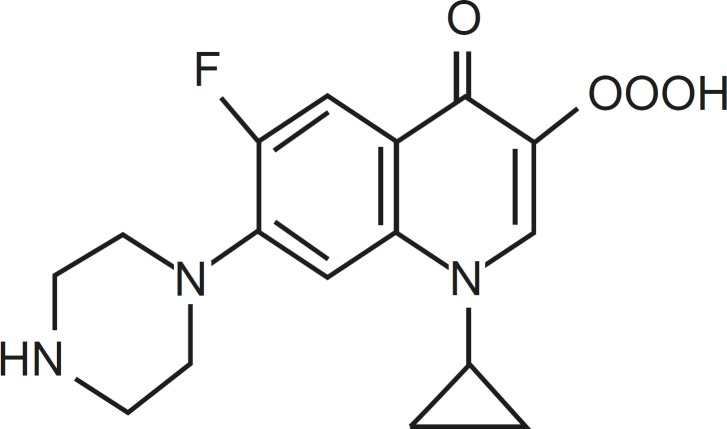
Structure of ciprofloxacin

Quinolones are a large and widely consumed class of synthetic drugs (1). First-generation (acidic) quinolones, including nalidixic acid and oxolinic acid, have been only used for treatment of urinary tract infections (2). However, modification of subsequent generations has increased their spectrum and potency. One of these modifications has been the addition of a fluorine atom at position C-6 of drug molecules, for instance in ciprofloxacin (CFX), which leads to wide potent activity against different Gram-negative bacteria (1). Moreover, due to the presence of a secondary amine in addition to carboxylic acid found in all members of family, CFX is a amphoteric quinolone rather than acidic one (Figure 1). 

Fluoroquinolones, such as CFX have been used to treat a great variety of infections, including urinary tract infections, blood stream infection, enteric infections or respiratory tract infections (3). Unfortunately, frequent use and sometimes misuse of CFX leads to the emergence of CFX-resistant bacteria, especially in Gram-negative bacteria such as *E. coli *(4). In *E. coli*, the major target for quinolones is DNA gyrase (2, 5, 6). DNA gyrase is a tetrameric enzyme composed of two A subunits and two B subunits encoded by *gyrA *and *gyrB*, respectively (7, 8). This enzyme belongs to type II topoisomerase family which is able to supercoil and to uncoil DNA helix by cleaving both strands of helix, passing another segment of the helix through resulting double strand break (DSB) and resealing this DSB in the expense of ATP hydrolysis (9). These activities are essential in DNA replication, transcription and recombination. On the other hand, there are some minor targets for quinolones in Gram-negative bacteria, including *parC *and *parE *encoding subunits of topoisomerase IV, another member of type II topoisomerase (2). Quinolones bind to gyrase-DNA complex, called cleavable complex due to the presence of DSB, and form gyrase-quinolone-DNA ternary complex (5, 6). Ultimate denaturation or disruption of gyrase in ternary complex results in the generation of DSB and thereby replication blockage and cell death (10, 11). 

Mutations in either *gyrA *or *gyrB *cause quinolone resistance. (12, 13). However, mutations in the *gyrA *gene are more common in quinolone-resistant clinical isolates of *E. coli *(14, 15). DNA sequence analysis has shown that most of the mutations have been located in the first half of *gyrA *gene in the region called quinolone resistance determining region (QRDR) (12). This region is in close relation with the active site of GyrA (Tyr-122), which interacts with DNA and quinolone (16). However, a mutation outside this region was also reported (17). To gain more information on mutations sites in *gyrA*, spontaneous CFX-resistant mutants of pathogenic *E. coli *ATCC 25922 and non pathogenic one, MG1655 were isolated on LB agar containing CFX. 

## Experimental

**Materials**

*Antimicrobial agents*

CFX was obtained from Bayer Corporation (West Haven, CT) as standard reference powder for laboratory use. Stock solution was 10 mg/ mL.

*Bacterial strains*

*E. coli *ATCC 25922 was purchased from Iran Research Organization for Science and technology. MG1655 was a laboratory strain of Prof. R. G. Lloyd. 

*Media*

The liquid medium used for bacterial growth was Luria broth (LB) (Merck, Germany), and the solid medium was LB supplemented with 1.5% agar (LBA) (Merck, Germany). 

**Methods**

*Antibiotic susceptibility test*

MICs of CFX for control strains, ATCC 25922 and MG1655, were determined using broth dilution method (18). For each strain, two independent cultures were grown for 24 h at 37°C in LB without CFX. Inocula of 10^6^ colony forming units (CFU) from each culture were inoculated in duplicate onto screw capped tubes containing different concentrations of CFX ranging from 5 ng/mL to 50 ng/mL in 8 mL LB broth. The MIC was defined as the lowest concentration of CFX which prevented any detectable growth after 24 h of incubation at 37°C. MICs for control strains were determined in three independent experiments. MICs for CFX-resistant clones isolated during isolation of CFX-resistant mutant (see below) were measured with the same conditions with higher concentrations of CFX. 

*Isolation of spontaneous CFX-resistant mutants from solid medium*

For each control strains, five independent cultures were grown overnight in LB without CFX (permissive medium). Viable cell counts in these cultures were determined by plating several dilutions on LBA without CFX. 150 μL samples from each overnight culture (containing approximately 10^8^ cells/mL) were spread in duplicate on LBA containing CFX (non-permissive medium). At 24 h intervals (totally for three days) visible colonies were counted and randomly picked up and cultured for determination of mutation frequency and MIC, respectively. 

*PCR amplication and DNA sequencing*

A single colony from each clone grown on LBA containing CFX was suspended in 100 μL of sterile water and then heated at 95°C for 3 min and cooled on ice. It can be used as a PCR template for *gyrA *fragment amplification with the *gyrA *specific primers, including forward 5΄- CTGAAGCCGGTACACCGT-3΄ and reverse 5΄- GGATATACACCTTGCCGC-3΄ primers (19). This region of the *gyrA *gene (577 bp) contains the QRDR. QRDR is a small region from amino acid 67 to amino acid 106 in GyrA (A subunit) (12). 

## Results

The MIC of CFX for control strains, including MG1655 (nonpathogenic) and *E. coli *ATCC 25922 were determined. They were 35 ng/mL and 9 ng/mL for MG1655 and *E. coli *ATCC 25922, respectively. These figures are consistent with those were previously obtained (20). 

To isolate spontaneous CFX-resistant mutants, MG1655 and *E. coli *ATCC 25922 were grown in permissive liquid medium and then plated on to LBA containing 40 ng/mL and 12 ng/mL CFX, respectively. The frequency of CFX-resistant mutation for *E. coli *ATCC 25922 was 10×10^-6^ mutant/total counts/day and for MG1655 was 40×10^-7^ mutant/total counts/day. These are consistent nearly with previous data (20). Five colonies were randomly selected from each plate, and totally 100 CFX-resistant colonies originated from both control strains were used for further investigation. 

The MICs of CFX for these clones were determined and presented in increasing order of resistance in Table 1. MICs of majority of clones, which were taken after a day of incubation and originated from MG1655, were 62.5 or 75 ng/mL (Table 1). Moreover, most of clones originated from *E. coli *ATCC 25922 and taken on the first day of incubation had MIC of 18 ng/mL (Table 1). However, MICs of clones formed on the third day of incubation were higher than above figures. This means that these clones may gain extra mutations following exposure to CFX. 

**Table 1:** Ciprofloxacin susceptibility of control strains and their derived mutants

**Strain **	**MIC** ^a^ ** (ng/mL) **
a) MG1655 wild type	35
W1-W22	62.5
W23-W43	75
W44-W46	125
W47-W48	312
W49-W50	625
b) *E. coli *ATCC 25922	9
P1-P32	18
P33-P43	75
P44-P46	312
P47-P48	625
P50	1250

^a^ Data are mean of at least two independent experiments

**Figure 2:**
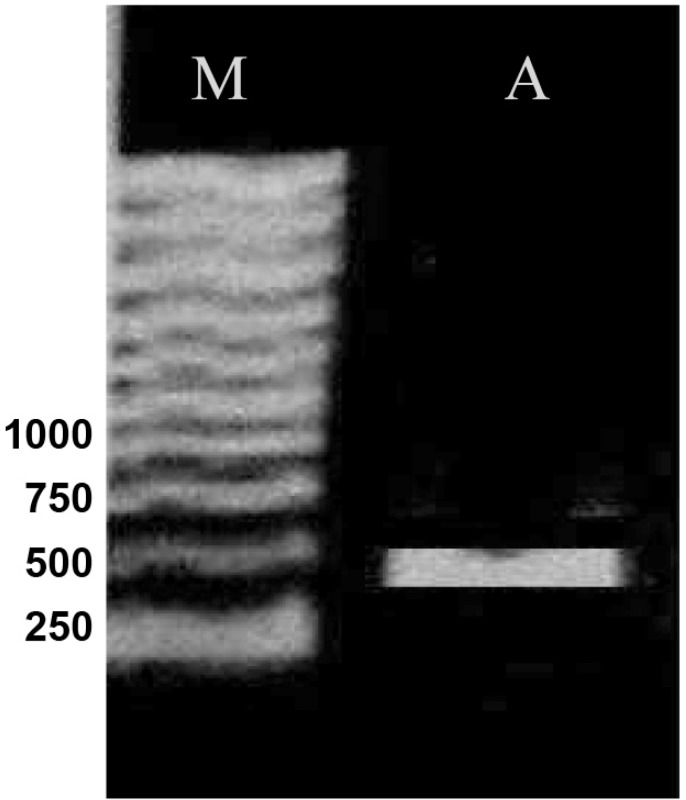
Gel analysis of PCR product. Lanes M and A contain 1 kb DNA ladder marker and PCR product, respectively

**Figure 3:**
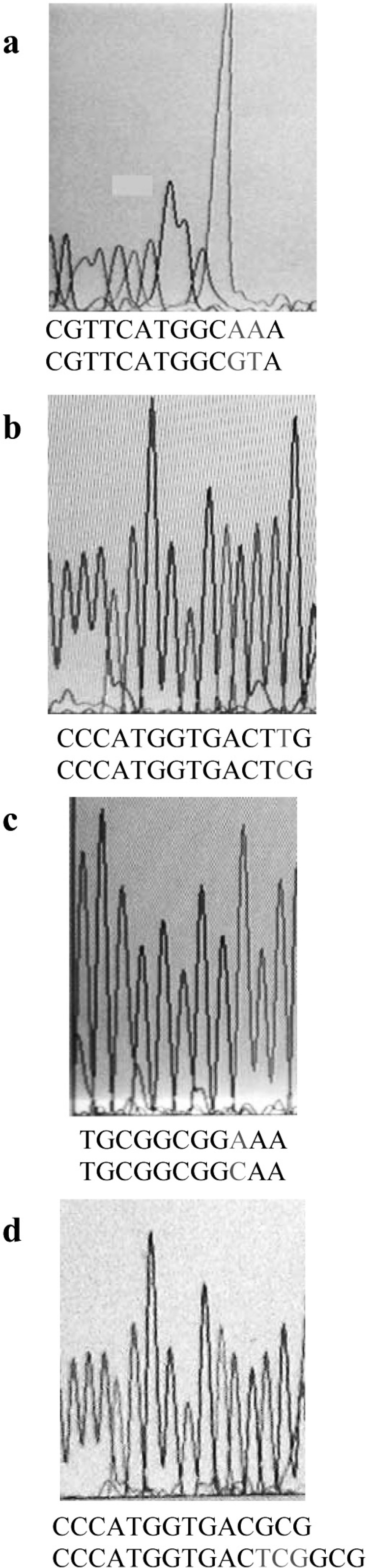
Sequence output from the PCR products of different CFX-resistant mutants, using forward or reverse primer. On the bottom of each graph, the first and second nucleotide sequences belong to mutant and wild type *gyrA*, respectively

On the basis of the MIC results, totally 50 clones from both genetic backgrounds, considering full range of MIC, were chosen for PCR analysis. Part of *gyrA *gene containing QRDR was amplified. Figure 2 shows the result of PCR amplification. The same results were obtained for all clones. Then, the PCR products were sequenced by using forward or reverse primer. Finally, these sequences were compared with the published *gyrA *sequence of *E. coli *K-12 by using DNA for windows software. Putative fluroquinolone resistance mutations were defined as nucleotide alterations, including substitutions, deletions, or insertions. The sequence comparison showed that CFX resistance property is associated with alterations in nucleotide sequence of *gyrA *gene (Figure 3). Nucleotide substitutions (Figure 3a, b, c) and deletion (Figure 3d) were seen in different sites of *gyrA *gene. These kinds of alterations, shown in Figure 3, were obtained from different clones.

Alterations in nucleotide sequence of gene would cause a change in the amino acid sequence of the encoding protein product. Table 2 shows the amino acid changes that were caused by nucleotide changes shown in Figure 3. Silent mutations are not presented. Forty five out of fifty clones carried mutations in *gyrA*. The remaining might have mutations in other CFX target sites in genes, such as *gyrB*, *parC *or *parE*. 

**Table 2:** Ciprofloxacin resistance mutations in the *gyrA *gene

**Strain **	**Mutation **
a) MG1655	Wild type
W1-W4	Ser-83→Leu (TTG)
W14	Tyr-50 (TAC)→Phe (TTT)
W23-W26	Wild type
W27	Tyr-50 (TAC)→Phe (TTT)
W28	Ser-83→deleted
W29-W38	Ser-83→Leu(TTG)
W43	Ala-119 (GCA)→Glu (GAA)
W44	Ser-83→Leu (TTG)
W47	Ser-83→Leu (TTG)
W49	Ser-83→Leu (TTG)
b) *E. coli *ATCC 25922	Wild type
P1	Wild type
P2-P11	Ser-83→Leu (TTG)
P33-P42	Ser-83→Leu (TTG)
P44	Ser-83→Leu (TTG)
P47-P49	Ser-83→Leu (TTG)
P50	Ser-83→Leu (TTG)

Of 45 clones harboring mutation in *gyrA*, 41 carried mutation altering amino acid 83. Amino acid change detected at this site was Ser to Leu. This kind of mutation has already been reported (14, 15). Moreover, in one out of 4 remaining clones the amino acid 83 was deleted. This shows the importance of this position in gain of CFX resistance. Clones containing the alteration of amino acid 83 had different MICs, suggesting that those with higher MICs probably had extra mutations in other genes containing CFX target sites. In addition, in two other non-83 codon alteration clones, there was a mutation that alters Tyr-50 to Phe. Occurrence of mutation outside of the QRDR region was reported, but it was at position 51 (17). On the other hand, in the last remaining clone, there was a mutation which altered Ala-119 to Glu. This codon is near Tyr-122 which is the active site of GyrA (16). This mutation has not been reported before.

## Discussion

The high potency, broad spectrum of activity and relative tolerability of the fluoroquinolones, like CFX have led to widespread use and misuse of these agents for different therapeutic purposes. These lead to rapid emergence of resistant strains to these agents. Fluoroquinolones target enzyme in Gram-negative bacteria, such as *E. coli *is DNA gyrase. Mutations in both *gyrA *and *gyrB *encoding subunits of DNA gyrase lead to resistance to these agents. The majority of resistant clinical isolates possess mutations in QRDR section of GyrA (14, 15). This study investigated the generation of spontaneous CFX-resistant mutants in cultures of *E. coli *strains with different genetic backgrounds. 

Our study showed that the frequency of CFX resistant mutants in cultures of *E. coli *strains is low in laboratory condition. However, some of isolated mutants had high MICs following exposure to CFX. This implies why misuse or inappropriate use of this drug could lead to rapid development of resistance in vivo. 

We found that most of CFX-resistant mutants contained mutations in QRDR section of GyrA at amino acid 83 without considering their genetic backgrounds (pathogenic or non pathogenic). It was suggested that the replacement of hydrophilic amino acid (Ser) by hydrophobic amino acid (Leu, Trp, Ala or Pro) at amino acid 83 leads to induction of a local conformation change of the A subunit (12). This implies the importance of this site in interaction of gyrase and CFX. Moreover, our finding that showed the deletion of amino acid 83 leads to resistance to CFX reconfirms the importance of this site and suggests that either substitution or deletion of this site leads to the loss of enzyme-drug interaction. 

These clones possessed different MICs of CFX. This reveals that the generation of a mutation in QRDR is the minimum necessity for resistance to CFX and gaining extra mutations in other CFX targets results in elevation of resistance. Furthermore, finding two clones with a mutation at position 50 outside the QRDR region, suggests the importance of more amino acids in the production of active site of DNA gyrase. 

Our finding that showed the alteration of Ala-119 to Glu alone causes resistance to CFX also suggests that a change from hydrophobic amino acid to acidic amino acid at amino acid 119 may have an effect on the conformation of the active site of enzyme (Tyr-122) and its interaction with neighboring amino acids and thereby the inability of CFX to bind to gyrase. In addition, for this clone having the same MIC as most of those with substitution at Ser-83 (75 ng/mL) indicates that position 119 may be as important as position 83 in making bacteria resistant to CFX. 

Briefly, the presence of mutations at amino acids 50 and 119 suggests that in addition to QRDR section and Tyr-122, these sites are also essential for DNA gyrase activity. 
